# Editorial Comment: Low-Intensity Shock Wave Therapy in Sexual Medicine-Clinical Recommendations

**DOI:** 10.1590/S1677-5538.IBJU.2020.01.09

**Published:** 2020-01-13

**Authors:** P Capogrosso, A Frey, CFS Jensen, G Rastrelli, GI Russo, J Torremade, M Albersen, I Gruenwald, Y Reisman, G Corona, Rodrigo R. Vieiralves

**Affiliations:** 1 Vita-Salute University Ospedale San Raffaele Department of Urology Department of Urology; Ospedale San Raffaele, Vita-Salute University, Milano, Italy; 2 University of Southern Denmark Department of Urology ^2^ Department of Urology, University of Southern Denmark, Esbjerg and Odense, Denmark; 3 University of Copenhagen Herlev and Gentofte Hospital Department of Urology Department of Urology, Herlev and Gentofte Hospital, University of Copenhagen, Copenhagen, Denmark; 4 University of Florence Department of Experimental, Clinical and Biomedical Sciences Andrology, Female Endocrinology and Gender Incongruence Unit Andrology, Female Endocrinology and Gender Incongruence Unit, Department of Experimental, Clinical and Biomedical Sciences, University of Florence, Florence, Italy; 5 Urology Section-University of Catania Urology Section-University of Catania, Catania, Italy; 6 Hospital Universitari de Bellvitge Hospital Universitari de Bellvitge, Barcelona, Spain; 7 University of Leuven Department of Development and Regeneration Laboratory of Experimental Urology Laboratory of Experimental Urology, Department of Development and Regeneration, University of Leuven, Leuven, Belgium; Department of Urology, University Hospitals of Leuven, Leuven, Belgium; University Hospitals of Leuven Laboratory of Experimental Urology; 8 Rambam Healthcare Campus Neuro-urology Unit Neuro-urology Unit, Rambam Healthcare Campus, Haifa, Israel; 9 Amstelland Hospital Men's Health Clinic Men's Health Clinic, Amstelland Hospital, Amsterdam, The Netherlands; 10 Maggiore-Bellaria Hospital Medical Department Endocrinology Unit Endocrinology Unit, Medical Department, Azienda USL, Maggiore-Bellaria Hospital, Bologna, Italy; 1 Serviço de Urologia, Hospital Federal da Lagoa, Rio de Janeiro, RJ, Brasil

## COMMENTS

In recent times, low-intensity shockwave therapy (LISWT) has become one of the therapeutic modalities for the treatment of andrological disorders with controversial findings, which makes it difficult to recommend them in the guidelines (1–3). In this scenario this interesting paper aimed to evaluate the applicability of the LISWT in the treatment of erectile dysfunction (ED), Peyronie's disease (PD) and chronic prostatitis/chronic pelvic pain syndrome (CP/CPPS).

During their review, the authors analyzed 11 RCTs and 5 meta-analyzes that investigated LISWT for ED, 4 RCTs and 1 meta-analysis for PD and 5 RCTs for CP / CPPS. For erectile dysfunction purposes, although there is a tendency in this direction, the review made clear a heterogeneity among treatment protocols, with controversial findings and indication being restricted to vasculogenic ED. Before starting treatment, patients should be aware that the scientific evidence is controversial and that the expected improvement may not be clinically relevant. In relation to PD, data available from RCTs is poor. Patient inclusion criteria vary from stable disease to non-stable disease and follow-up assessment varies too much (24 weeks to 1 year) using different sources of energy and heterogeneous protocols proposed, making any comparison difficult. Nevertheless, in a large prospective, randomized, double-blind, placebo-controlled study, with four weekly treatment sessions of ESWT, they observed a significant improvement in penile pain and thus LISWT could be an option for this purpose (4, 5). However, patients should be counseled that no effect can be expected on curvature and plaque size. In the same direction we have the results obtained for the CP/CPPS. There is no evidence for maintenance of the improvement over time. LISWT could be applied in patients with CP / CPPS, especially to non-responders to conventional therapies but again, patients should be advised about the lack of robust evidence with long- term shockwave therapy.

Despite the great enthusiasm and effort to demonstrate LISWT effectiveness in treating ED, PD and CP/CPPS, data over time are not robust and several uncertainties like if it is indeed an effective treatment, what is the best protocol to ensure a higher probability of treatment success and how long does the effect last, still persist. We are on the way, but no doubt future studies are still needed to address these questions.
